# Supporting endocrine therapy adherence in women with breast cancer: findings from the ROSETA pilot fractional factorial randomized trial

**DOI:** 10.1093/abm/kaaf003

**Published:** 2025-01-31

**Authors:** Samuel G Smith, Sophie M C Green, Emma McNaught, Christopher D Graham, Robbie Foy, Pei Loo Ow, David P French, Louise H Hall, Hollie Wilkes, Christopher Taylor, BA, Rachel Ellison, Erin Raine, Rebecca Walwyn, Daniel Howdon, Jane Clark, Nikki Rousseau, Jacqueline Buxton, BA, Sally J L Moore, Jo Waller, Catherine Parbutt, Galina Velikova, Amanda Farrin, Michelle Collinson

**Affiliations:** Leeds Institute of Health Science, University of Leeds, Leeds, LS2 9NL, United Kingdom; Leeds Institute of Health Science, University of Leeds, Leeds, LS2 9NL, United Kingdom; Clinical Trials Research Unit, Leeds Institute of Clinical Trials Research, University of Leeds, Leeds, LS2 9NL, United Kingdom; Department of Psychological Sciences and Health, University of Strathclyde, Glasgow, G1 1QE, United Kingdom; Leeds Institute of Health Science, University of Leeds, Leeds, LS2 9NL, United Kingdom; Clinical Trials Research Unit, Leeds Institute of Clinical Trials Research, University of Leeds, Leeds, LS2 9NL, United Kingdom; Division of Psychology and Mental Health, University of Manchester, Manchester, M13 9PL, United Kingdom; Leeds Institute of Health Science, University of Leeds, Leeds, LS2 9NL, United Kingdom; Clinical Trials Research Unit, Leeds Institute of Clinical Trials Research, University of Leeds, Leeds, LS2 9NL, United Kingdom; Clinical Trials Research Unit, Leeds Institute of Clinical Trials Research, University of Leeds, Leeds, LS2 9NL, United Kingdom; Clinical Trials Research Unit, Leeds Institute of Clinical Trials Research, University of Leeds, Leeds, LS2 9NL, United Kingdom; Leeds Institute of Health Science, University of Leeds, Leeds, LS2 9NL, United Kingdom; Clinical Trials Research Unit, Leeds Institute of Clinical Trials Research, University of Leeds, Leeds, LS2 9NL, United Kingdom; Leeds Institute of Health Science, University of Leeds, Leeds, LS2 9NL, United Kingdom; Department of Clinical and Health Psychology, Leeds Teaching Hospitals NHS Trust, Leeds, LS9 7TF, United Kingdom; Clinical Trials Research Unit, Leeds Institute of Clinical Trials Research, University of Leeds, Leeds, LS2 9NL, United Kingdom; Independent, Leeds, LS2 9NL; Independent, Leeds, LS2 9NL; Wolfson Institute of Population Health, Queen Mary University of London, London, E13 8SP, United Kingdom; Medicines Management and Pharmacy Services, Leeds Teaching Hospitals NHS Trust, Leeds, LS9 7TF, United Kingdom; Leeds Institute for Medical Research, University of Leeds, Leeds, LS9 7TF, United Kingdom; Clinical Trials Research Unit, Leeds Institute of Clinical Trials Research, University of Leeds, Leeds, LS2 9NL, United Kingdom; Clinical Trials Research Unit, Leeds Institute of Clinical Trials Research, University of Leeds, Leeds, LS2 9NL, United Kingdom

**Keywords:** breast cancer, optimization, medication adherence, acceptance and commitment therapy, text messaging, factorial trial

## Abstract

**Background:**

Adherence to adjuvant endocrine therapy (AET) in women with breast cancer is poor. Multicomponent intervention packages are needed to address adherence barriers. Optimizing these packages prior to definitive evaluation can increase their effectiveness, affordability, scalability, and efficiency.

**Purpose:**

To pilot procedures for an optimization-randomized controlled trial (O-RCT) of the 'Refining and Optimizing Strategies to support Endocrine Therapy Adherence' (ROSETA) intervention.

**Methods:**

This was a multisite individually randomized external pilot trial using a 2^4-1^ fractional factorial design (ISRCTN10487576). Breast cancer survivors prescribed AET were recruited from 5 hospitals and randomized to one of 8 conditions, each comprising a combination of 4 intervention components set to “on” or “off” (SMS messages, information leaflet, guided self-help, and self-management website). We set criteria to inform the decision to progress to an O-RCT for consent rate, component adherence, and availability of outcome measures, with predefined cutoffs for “green” (proceed), “amber” (minor changes), and “red” (major changes).

**Results:**

Among 141 eligible patients, 54 (38.3%) consented (green range). At least 50.0% of participants adhered to the minimum threshold set for each intervention component (green range). Data for one of the 3 medication adherence measures were available (amber range). Most (86.8%) participants were satisfied with their trial experience. Exploratory analysis indicated some evidence of a negative main effect of the information leaflet on medication adherence (adjusted mean difference = 0.088, 95% CI, 0.018, 0.158).

**Conclusions:**

Progression to a fully powered O-RCT of the ROSETA intervention package is feasible, but review of medication adherence measures is required.

## Teaser

A pilot optimisation trial of a multi-component behavioral intervention to support medication adherence in women with breast cancer demonstrated a fully powered optimisation trial is feasible.

## Introduction

Breast cancer is the most common cancer worldwide, and the incidence is rising.^1–3^ In the United Kingdom, there are around 55 900 new cases of breast cancer annually and approximately 11 500 deaths per year.^4^ Around 80% of all breast cancers are hormone receptor-positive (HR+) tumors.^5^ Standard practice in high-income countries, including the United States and United Kingdom, is to offer women with early stage HR + tumors adjuvant endocrine therapy (AET) to reduce risk of recurrence.^6,7^

Recent evidence suggests 7-8 years of AET, including at least 5 years of an aromatase inhibitor (eg, anastrozole, letrozole, exemestane) could be optimal to balance efficacy and side effects.^8,9^ However, women prescribed AET often have low medication adherence despite this increasing the risk of recurrence and all-cause mortality.^6,10–13^ Among women who initiate AET, up to 3-quarters do not take it as prescribed,^10,14–17^ with adherence decreasing across the first 5 years of use.^16,18^ The sharpest decrease in AET adherence is in the first year of use, indicating a suitable period in which to provide additional support.^19–22^

A number of systematic reviews have synthesized the evidence on barriers to AET adherence, and include both modifiable and nonmodifiable factors, indicating a multicomponent intervention is necessary.^23–25^ In the early stages of intervention mapping, we identified the most common potentially modifiable factors associated with nonadherence in women using AET, including experience of side effects (eg, hot flushes, night sweats), negative beliefs about the medication, psychological distress, forgetfulness, and low social support and self-efficacy.^26^ In subsequent stages of intervention mapping, we prioritized targeting 4 of these barriers: forgetting, medication beliefs, psychological distress, and medication side effects (Supplementary Material 1). Prior to evaluating the effect of an intervention that addresses these targets, here we report the findings of a pilot trial that assessed the feasibility of undertaking a larger trial to optimize this intervention package.

Interventions aiming to support AET adherence have often been unsuccessful, possibly due to a tendency to focus only on increasing knowledge.^27–29^ The most comprehensive meta-analysis of AET adherence interventions, including 25 studies and 367 873 women, showed interventions have a positive effect overall.^29^ However, except for educational interventions, which were ineffective, and lowering medication costs, which was consistently effective, there was little insight into the most useful components of multicomponent interventions.

The collective lack of understanding of how multicomponent interventions work could inhibit scientific progress. There have been calls for further experimental research investigating the mechanisms of multicomponent AET interventions.^29^ This approach is advocated by the multiphase optimization strategy (MOST), an engineering-inspired framework used to develop, optimize, and evaluate multicomponent interventions.^30^ MOST provides guidance on the use of highly efficient experimental designs to build multicomponent interventions that balance effectiveness against constraints such as affordability, scalability, efficiency, and equity.^31,32^

In line with the MOST framework, we used Intervention Mapping to prepare a theoretically informed multicomponent intervention to support AET adherence in women with early stage breast cancer (Supplementary Material 1).^26^ The 4 components include SMS messages to promote medication-taking habits,^33^ an information leaflet to promote balance in necessity beliefs versus concerns regarding AET,^34^ an adapted guided self-help intervention informed by Acceptance and Commitment Therapy (ACT) to enhance psychological flexibility and reduce psychological distress,^35^ and access to a website containing information about strategies for self-managing mild to moderate medication side effects.^36^

Prior to optimizing the package of intervention components, uncertainties regarding the feasibility of undertaking an optimization-randomized controlled trial (O-RCT) needed to be addressed. We therefore undertook a pilot trial to inform the decision of whether to conduct a fully powered O-RCT. The following objectives are addressed in this article: (1) establish eligibility, recruitment, retention, and follow-up rates; (2) establish intervention component adherence; (3) establish the availability and feasibility of collecting outcome and process data; (4) estimate the variability of planned outcome measure(s) and explore signals of efficacy; (5) estimate the cost of delivering each intervention component; and (6) describe the experience of participating in the trial. The protocols for this pilot O-RCT and data regarding the acceptability of the intervention components are reported elsewhere.^37–39^

## Methods

### Pilot trial design

This multisite exploratory pilot trial used a 2^4-1^ fractional factorial design with a nested mixed-methods process evaluation to be reported elsewhere.^38,39^ Women with early breast cancer were randomized with an equal chance of being allocated to one of 8 experimental conditions, with all participants receiving usual care (Table 1). Each candidate component was operationalized as a factor with 2 levels (on/off). A fractional factorial design halved the number of experimental conditions required compared to a full 2^4^ factorial design (16 experimental conditions), meaning all effects were aliased with other effects (Supplementary Material 2). As decision-making about an optimized intervention was not an aim of this pilot trial, aliasing of effects that occur in a fractional factorial design was not considered problematic. Follow-up was at 2- and 4 months postrandomization. The trial adheres to the CONSORT extension for pilot and feasibility trials^40^ and the candidate intervention components are described using the TIDieR checklist.^26,41^ The trial has been approved by Wales Research Authority Research Ethics Committee 3 (21/WA/0322).

**Table 1:** | 2^4-1^ Fractional factorial design used within the ROSETA pilot trial.

Condition	Usual Care	SMS	Leaflet	ACT	Website
1	Yes	Yes	Yes	Yes	Yes
2	Yes	Yes	Yes	No	No
3	Yes	Yes	No	Yes	No
4	Yes	Yes	No	No	Yes
5	Yes	No	Yes	Yes	No
6	Yes	No	Yes	No	Yes
7	Yes	No	No	Yes	Yes
8	Yes	No	No	No	No

### Participants

We recruited UK-based adult women affected by early stage (1-3a) breast cancer who had completed their hospital-based treatment in the past 12 months and were currently prescribed AET. Participants were recruited between May 20, 2022 and December 13, 2022. Full eligibility criteria are reported elsewhere.^37^ Participants were identified via 3 routes: (1) a research nurse based at the hospital site screened patient records prior to clinic visits; (2) patients who had self-referred to their care team to discuss medication side effects or adherence problems were identified; (3) a research nurse at the hospital site searched records for patients who had completed treatment in the past 12 months.

Anonymized data on age, ethnicity, staging, tumor type, and whether a patient was randomized were collected on a screening form for all potential participants. The reason for ineligibility or declining participation was recorded. For interested patients, a research nurse confirmed eligibility and recorded informed consent.

### Randomization

Following confirmation of informed consent, registration, confirmation of eligibility, and completion of the baseline questionnaire, the research nurse notified the University of Leeds Clinical Trials Research Unit (CTRU) that the patient could be randomized. Randomization was performed by an authorized member of the CTRU using the CTRU automated randomization system and participants were allocated to one of the experimental conditions (Table 1). Stratified permuted block randomization ensured experimental conditions were balanced for the stratification factor (recruitment route) and to ensure the design was close to orthogonal. Randomization lists produced by the trial statistician were held securely within the CTRU with access restricted to authorized individuals. The research nurse was informed of the randomization result via email and was asked to send an intervention summary sheet describing a timeline for receipt of the allocated intervention components.

### Blinding

Blinding to randomized allocation was not possible for participants, therapists, research nurses, participants’ GPs or CTRU staff (including the Chief Investigator).

### Usual care

All participants received usual care, which was the standard care offered by sites to patients at this stage of their breast cancer treatment. Typically, this would include an end of treatment summary meeting with a breast care nurse but care could vary by site.

### Candidate intervention components

The full intervention development process is described elsewhere.^26^

#### SMS *m*essages

A series of 43 brief text messages were sent over a period of 4 months, aiming to support the establishment of medication-taking habits. The messages were co-developed with behavior change experts and women with breast cancer in a series of quantitative and qualitative studies.^26,33^ The messages targeted 6 behavior change techniques theorized to support habit formation, from version 1 of the BCT taxonomy^42–46^: habit formation, prompts/cues, restructuring the physical environment, adding objects to the environment, action planning, and self-monitoring of behavior. Messages were sent directly to participant cell phones from an automated system, commencing up to 1-week after randomization. Messages were sent daily for 2 weeks, twice weekly for 8 weeks, and then weekly for 6 weeks,^43,47,48^ in addition to 3 opening messages, a closing message, and a monthly message reminding participants they could opt out.

#### Information leaflet

A 6-page information leaflet designed to increase perceived necessity and reduce concerns regarding AET was emailed to participants by the research site immediately after randomization. The leaflet was designed with input from women taking AET for breast cancer and was informed by the Necessity Concerns framework and Common-Sense Model of Self-regulation.^26,49,50^ It included diagrams explaining how AET works, information about the benefits of taking AET and the prevalence of potential side effects, answers to common concerns, and quotes from women taking AET about their motivations to take the medication.

#### ACT guided self-help

An ACT-guided self-help intervention aimed to increase psychological flexibility and reduce psychological distress. The intervention was adapted from an existing program tested in people with muscle disorders.^35^ The component consisted of 4 modules, covering 4 ACT-based skills, including mindfulness, unhooking, following values, and living beyond labels. Each module comprised a module booklet, audio files, and home practice tasks. The modules were supplemented by 5 therapist-led sessions: a 15-min introduction, three 25-min sessions following modules 1, 2, and 3, and a 15-min closing session following module 4. During sessions, the therapist reflected on the previous module content and participant’s use of skills and introduced the subsequent module. Sessions were recommended to take place weekly, commencing within 4 weeks of randomization.

#### Self-management website

A website to support women to self-manage side effects experienced from AET was developed based on recommendations from women with breast cancer and healthcare professionals.^51^ The website was informed by an umbrella review of side-effect self-management strategies^36^ for the most common side effects, including joint pain, vulvovaginal symptoms, hot flushes, gastrointestinal symptoms, sleep difficulties, and fatigue. Ratings of the scientific evidence for each management strategy were provided to summarize the strength of evidence. The website also included videos from women taking AET and signposting to further information and support. Log-in details to the website were sent to the participant by the research site immediately after randomization.

### ACT therapists

Therapists were Health and Care Professions Council or UK Council for Psychotherapy (UKCP) registered psychologists or psychotherapists (band 7a or above). Twelve therapists received 2 half-days of training from a clinical psychologist (CG) with expertise in ACT, including general teaching about ACT and practice of intervention-specific therapy methods. Ongoing fortnightly group supervision (60 min) was offered throughout the trial, delivered by CG. ACT therapists were able to access local clinical supervision as part of standard clinical practice. Therapists were allocated to patients nonrandomly based on therapist availability.

### Measures

Participant reported outcome measures were collected online at baseline, 2- and 4 months postrandomization. Nonrespondents were reminded by telephone, email, and/or SMS. Baseline data were collected on name, postcode, NHS number, email address, telephone number, date of birth, gender, marital status, employment, education, menopausal status, stage of cancer at diagnosis, breast cancer treatment received, comorbidities, and AET regimen.

The primary outcomes were: participant consent rate (source: screening and recruitment data) component adherence and availability of medication adherence measures (source: participant self-report and by research teams across the 4-month follow-up period) (Table 2).

**Table 2:** | Progression criteria to inform decision for progressing to O-RCT.

	Green	Amber	Red
Eligible patients consent rate	≥30%	≥10%	<10%
Component adherence			
75% of SMS messages received with no opt out	≥50%	≥20%	<20%
Read “at least some” of the information leaflet	≥50%	≥20%	<20%
Completed 2/4 ACT modules	≥50%	≥20%	<20%
Registered and logged onto website at least once	≥50%	≥20%	<20%
Availability of adherence measures with ≥75% complete data	≥2	≥1	0

Green (go): optimization phase is feasible with no changes to design or procedures; Amber (modify): optimization phase is feasible following minor enhancement of procedures; Red (stop): optimization phase is not feasible without major changes.

Component adherence was assessed based on the minimum dose of each intervention component anticipated to demonstrate an effect. Routinely collected SMS sent receipts were obtained, along with opt out messages sent by participants to calculate the proportion of participants who received >75% of SMS messages with no opt out. Participants were asked how much of the information leaflet they read (“None of it” “at least some” “all of it”). Therapist’s report assessed the proportion of participants meeting the progression criterion of completing 2/4 ACT modules, defined as the participant attending the session associated with the module and perceived to have engaged with “at least some of” the module materials. Adherence to the guided self-help component was also assessed through participant self-report of how much of the home practice tasks they completed for modules 1–3 (“I didn’t do any of the home practice,” “I did at least some of the home practice,” “I did all of the home practice”). Analytics data was used to compute the proportion of participants who registered (yes/no) and logged onto the website at least once (yes/no).

Patient-reported outcome measures at baseline, 2- and 4 months assessed medication adherence (Voils DOSE nonadherence measure-extent scale^52^ [whereby a higher score represents more nonadherence], Morisky Medication Adherence Scale [MMAS]), quality of life (European Organization for Research and Treatment of Cancer QLQ-C30,^53^ -BR45,^54^ -IL133, EQ-5D-5L,^55^ McGill QoL-revised^56^), psychological flexibility (Multidimensional psychological flexibility inventory-short form^57^), beliefs about medication (Beliefs about medicines questionnaire-AET^58^), habit formation (Self-Report Behavioral Automaticity Index^59^) and psychological distress (Depression anxiety stress scales^60^).

Trial experience data were collected at 4 months via participant self-report (Study participant feedback questionnaire^61^) and qualitative interview. Usual care data were collected via participant self-report (at 2- and 4 months) and by site research teams (at 4 months).

We planned to obtain individual-level NHS prescribing and/or dispensing data for each participant for the duration of the trial. Intervention component costs were estimated using the Schedule of Events Cost Attribution Tool (SoECAT) and Personal Social Sciences Research Unit costs (PSSRU). Safety and fidelity data were collected by research teams throughout the follow-up period. Full details of the outcome assessments are described in detail elsewhere.^37^

### Sample size

We estimated that a sample of 80 participants (*n* = 10 per condition), allowing for 80% retention,^28^ would be sufficient to inform the sample size of the optimization trial, assuming adherence data are pooled across conditions.^62^

### Statistical analysis

Analysis was undertaken on the intention-to-treat population and focused on descriptive statistics and confidence interval estimation rather than formal hypothesis testing, and therefore no *P*-values were reported. Outcome measures were scored according to relevant scoring manuals with missing item-level data handled according to guidance where available or imputed using the half-rule.^63^ The decision on whether to proceed to a fully powered O-RCT was informed by predefined progression criteria (Table 2). Progression criteria were developed based on relevant existing literature and consensus from the trial management group.^64^ They were approved by the independent trial steering committee, who had oversight of the trial delivery. Meeting the green threshold indicates the O-RCT can proceed with no changes needed, the amber threshold indicates minor changes are needed prior to proceeding, and the red threshold indicates major changes are needed prior to proceeding to the O-RCT or not proceeding to an O-RCT at all. The patient consent rate was calculated using the number of eligible patients as the denominator. Binary variables were calculated for each intervention component to indicate whether a participant had met the definition of complete. The proportion of participants meeting this definition was calculated for each component. Digital prescribing and dispensing data are not currently accessible for research purposes therefore this measure will be classified as unobtainable for the progression criteria. We ceased use of the MMAS-8, as the key validation paper has since been withdrawn. A binary variable was calculated for each participant to determine the level of completion of the Voils Dose nonadherence measure—extent scale.

Although underpowered, proof of principle was explored by investigating between-group change in outcomes (medication adherence, quality of life, symptoms and side effects, medication beliefs, habits, psychological distress, and psychological flexibility) via linear regression models. Two- and 4-month outcomes were analyzed separately. We interpreted effects with *P* < .05 as indicating some evidence of a main effect, and effects with *P* < .15 as indicating a trend. Point estimates, adjusted for the stratification factor and baseline score, were calculated for the main effects and interaction effects for all medication adherence, quality of life, and process variables in which a change is hypothesized. Coefficients for all effects are reported as they originate from the regression model, which is half what they would traditionally be defined to be, due to the use of effect coding (−1, +1). As the 2^4-1^ design did not include all possible combinations of factor levels, all effects were aliased with other effects (Supplementary Material 2). We therefore focused our exploratory analysis on the main effects of components. We planned to estimate the intraclass correlation of the therapist effect, however too few therapists were trained for this to be calculated. All analyses were conducted in SAS, version 9.4.

## Results

### Screening, recruitment, and retention

**Figure 1.**
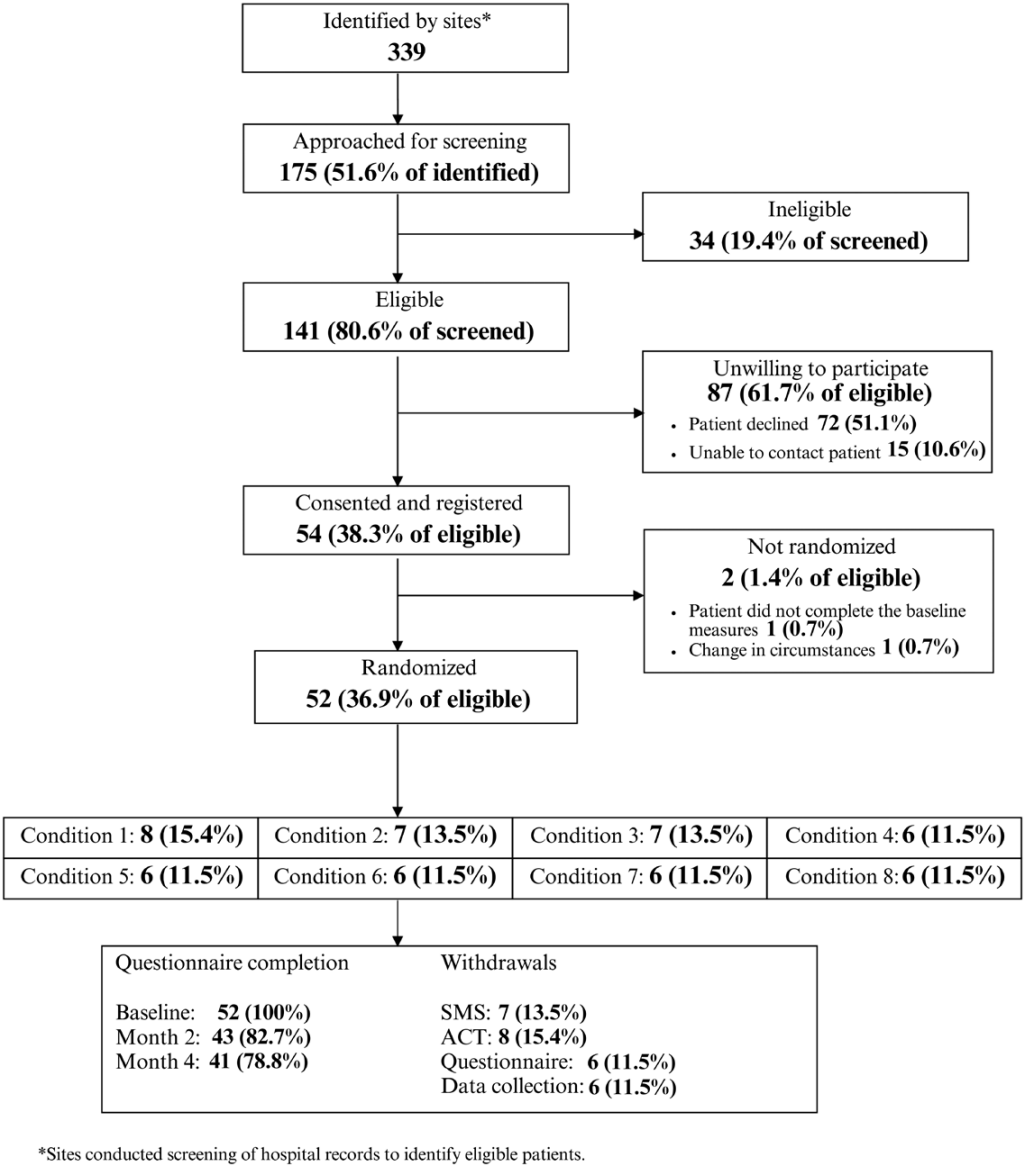
CONSORT flow.

Recruitment took place across 5 sites. Three hundred thirty-nine patients were identified, and 175 (51.6%) were approached for screening of whom 141 (80.6%) were eligible (Figure 1). The most common reasons for ineligibility included not being able to access a mobile phone to receive SMS messages (30.8%), not willing to receive frequent SMS messages (30.8%) and not being able to access a computer or smart device (30.8%). Of eligible patients, 54 (38.3%) consented to registration, and 52 (36.9%) were randomized, meeting the green progression criterion. Among the 87 patients who were eligible but did not consent, 15 (17.2%) could not be contacted and 72 (82.8%) declined. The most common, nonmutually exclusive reasons for declining were personal circumstances (15.3%, 11/72), not needing additional support (11.1%, 8/72), not interested in taking part (9.7%, 7/72) and not having time to take part (9.7%, 7/72). Randomized participants were similar to those screened although some differences were noted for stage of cancer at diagnosis and recruitment route (Supplementary Material 3). Table 3 reports the baseline characteristics of randomized participants.

**Table 3:** | Characteristics of participants randomized, by component.

*N* (%)	Total sample (*n* = 52)	SMS (*n* = 28)	Information leaflet (*n* = 27)	ACT (*n* = 27)	Website (*n* = 26)
**Age**					
Mean (SD)	55.2 (10.8)	52.5 (12.4)	56.1 (12.1)	55.4 (11.0)	54.1 (12.0)
**Ethnic origin**					
White	45 (86.5)	25 (89.3)	23 (85.2)	24 (88.9)	22 (84.6)
Mixed	2 (3.8)	1 (3.6)	2 (7.4)	0 (0)	1 (3.8)
Asian	2 (3.8)	1 (3.6)	1 (3.7)	2 (7.4)	2 (7.7)
Black	3 (5.8)	1 (3.6)	1 (3.7)	1 (3.7)	1 (3.8)
**Marital status**					
Married	32 (61.5)	16 (57.1)	16 (59.3)	17 (63.0)	15 (57.7)
Living with a partner	5 (9.6)	4 (14.3)	2 (7.4)	3 (11.1)	3 (11.5)
Single	6 (11.5)	3 (10.7)	3 (11.1)	3 (11.1)	3 (11.5)
Divorced or separated	7 (13.5)	4 (14.3)	5 (18.5)	3 (11.1)	4 (15.4)
Widowed	2 (3.8)	1 (3.6)	1 (3.7)	1 (3.7)	1 (3.8)
**Education level**					
Postgraduate qualification	7 (13.5)	5 (17.9)	5 (18.5)	4 (14.8)	4 (15.4)
Degree level education	10 (19.2)	7 (25.0)	4 (14.8)	4 (14.8)	3 (11.5)
Higher educational qualifications (below degree level)	12 (23.1)	5 (17.9)	6 (22.2)	7 (25.9)	6 (23.1)
Vocational Qualifications (NVQ1 + 2)	6 (11.5)	3 (10.7)	4 (14.8)	3 (11.1)	4 (15.4)
A-Level or equivalent	5 (9.6)	2 (7.1)	3 (11.1)	2 (7.4)	3 (11.5)
GCSE/ O-Level/ CSE	11 (21.2)	6 (21.4)	5 (18.5)	6 (22.2)	5 (19.2)
No formal qualifications	1 (1.9)	0 (0)	0 (0)	1 (3.7)	1 (3.8)
**Employment status**					
Full time	22 (42.3)	9 (32.1)	9 (33.3)	13 (48.1)	9 (34.6)
Part time	9 (17.3)	7 (25.0)	6 (22.2)	2 (7.4)	3 (11.5)
Not currently working	9 (17.3)	5 (17.9)	3 (11.1)	6 (22.2)	6 (23.1)
Other	12 (23.1)	7 (25.0)	9 (33.0)	6 (22.2)	8 (30.8)
**Number of children**					
0	10 (19.2)	7 (25.0)	7 (25.9)	6 (22.2)	8 (30.8)
1+	42 (80.8)	21 (75.0)	20 (74.1)	21 (77.8)	18 (69.2)
**Menopausal status**					
Premenopausal	12 (23.1)	10 (35.7)	8 (29.6)	5 (18.5)	7 (26.9)
Peri-menopausal	3 (5.8)	2 (7.1)	0 (0.0)	2 (7.4)	2 (7.7)
Postmenopausal	30 (57.7)	11 (39.3)	17 (63.0)	15 (55.6)	15 (57.7)
Unsure	7 (13.5)	5 (17.9)	2 (7.4)	5 (18.5)	2 (7.7)
**Stage of cancer at diagnosis**					
Stage IA	19 (38.0)	8 (30.8)	12 (44.4)	9 (36.0)	7 (26.9)
Stage IB	2 (4.0)	0 (0.0)	1 (3.7)	1 (4.0)	0 (0.0)
Stage IIA	15 (30.0)	11 (42.3)	7 (25.9)	7 (28.0)	11 (42.3)
Stage IIB	8 (16.0)	4 (15.4)	2 (7.4)	4 (16.0)	4 (15.4)
Stage IIIA	6 (12.0)	3 (11.5)	5 (18.5)	4 (16.0)	4 (15.4)
Missing	2	2	0	2	0
**Current hormone therapy regimen**					
Tamoxifen	12 (23.1)	9 (32.1)	5 (18.5)	7 (25.9)	5 (19.2)
Aromatase inhibitor	40 (76.9)	19 (67.9)	22 (81.5)	20 (74.1)	21 (80.8)
**Breast cancer treatment received**					
Lumpectomy	43 (82.7)	23 (82.1)	23 (85.2)	26 (96.3)	20 (76.9)
Unilateral mastectomy	5 (9.6)	3 (10.7)	1 (3.7)	0 (0.0)	4 (15.4)
Bilateral mastectomy	2 (3.8)	1 (3.6)	2 (7.4)	1 (3.7)	2 (7.7)
Neoadjuvant chemotherapy	5 (9.6)	4 (14.3)	3 (11.1)	4 (14.8)	5 (19.2)
Adjuvant chemotherapy	18 (34.6)	10 (35.7)	8 (29.6)	13 (48.1)	9 (34.6)
Adjuvant radiotherapy	43 (82.7)	23 (82.1)	22 (81.5)	23 (85.2)	20 (76.9)
Monoclonal antibody-based therapy	4 (7.7)	1 (3.6)	1 (3.7)	3 (11.1)	3 (11.5)
Other	13 (25.0)	8 (28.6)	8 (29.6)	7 (25.9)	3 (11.5)
**Charlson Comorbidity Index**					
0	45 (86.5)	25 (89.3)	23 (85.2)	23 (85.2)	23 (88.5)
1	6 (11.5)	2 (7.1)	4 (14.8)	4 (14.8)	2 (7.7)
4	1 (1.9)	1 (3.6)	0 (0.0)	0 (0.0)	1 (3.8)

All participants (52/52) provided baseline data, 82.7% (43/52) at 2 months and 78.8% (41/52) at 4 months. Twelve (23.1%, 12/52) participants withdrew or were withdrawn from at least one trial process, with some withdrawing from multiple processes. Two participants were withdrawn from the ACT component for clinical reasons; one was inappropriately randomized as they breached an exclusion criterion (current receipt of psychotherapy), and another had an escalation of mental health difficulties. Eight participants withdrew from the ACT component. One withdrew before any ACT sessions, 3 between sessions 1 and 2, 3 between sessions 2 and 3, and one after session 4. Seven participants withdrew from the SMS component, all after receipt of at least some messages. Six participants withdrew from completing questionnaires and 6 withdrew from medical record data collection.

### Component adherence

Adherence for all intervention components met the green progression. Of the participants randomized to receive each component, 75.0% (21/28) received >75% of SMS messages with no opt out, 63.0% (17/27) read “at least some” of the leaflet, 63.0% (17/27) completed 2/4 ACT modules, and 73.1% (19/26) registered and logged onto the website at least once.

### Trial experience

At baseline, most participants either agreed (30.8%, 16/52) or strongly agreed (63.5%, 33/52) the information given to them prior to joining the trial was everything they wanted to know. Similar proportions either agreed (28.8%, 15/52) or strongly agreed (65.4%, 34/52) this information was easy to understand. At 2-month follow-up, all respondents (100%, 43/43) felt the time taken to collect data was acceptable, the impact on daily activities was acceptable (97.7%, 42/43 responded “yes”) and the way in which data were collected was acceptable (100%, 43/43). At 4 months, most participants (78.9%, 30/38) felt the overall commitment required for the trial was similar to what they expected; 13.1% (5/38) felt it was more than expected and 7.9% (3/38) felt it was less than expected. The majority agreed (50%, 19/38) or strongly agreed (36.8%, 14/38) that they were satisfied with their overall trial experience.

### Intervention costs

Table 4 shows a breakdown of the per participant cost of delivering each intervention component: SMS messages (£4.20, ~$5.36 [currency conversion, March 22, 2024]), information leaflet (£6.00, ~$7.66), guided self-help (£285.49, ~$364.58) and self-management website (£9.98, ~$12.74). A proportion of the costs associated with the self-management website are fixed costs (one-off annual fees for hosting the website and updating the information) and therefore an increase in overall number of participants would decrease the cost per participant.

**Table 4:** | Per participant cost of delivering ROSETA intervention components and the source of cost data.

	Items	Source	Per participant cost
SMS messages	Sending SMS messages	SoECAT	£4.20
Information leaflet	Email leaflet by research nurse (assumes 10 min)	SoECAT	£6.00
Guided self-help	Band 7 Clinical Psychologist 5 sessions, assumed total 3 h and 50 min Book session (Band 4) (10 min) Total cost	PSSRU^a^ PSSRU^b^	£279.83 £5.66 £285.49
Website	Email log-in details by research nurse (assumes 10 min) Website hosting and security certificate and registration of domain name Website updating (text only) Est. 4 h per year, research fellow grade 7 (SP 36) Total cost	SoECAT Investigators Investigators	£6.00 £71.00 (one-off fee), £1.37 (per participant) £135.56 (one-off fee), 2.61 per participant) £9.98

^a^This cost used estimates for a registrar grade hospital doctor as this is a similar band and provides the best estimate of hourly overhead and indirect costs available.

^b^This cost used estimates for a band 4 professional staff as this is an equivalent band and provides the best estimate of hourly overhead and indirect costs available.

Abbreviations: SoECAT, schedule of events cost attribution tool; PSSRU, Personal social services research unit.

### Signals of effectiveness

Supplementary Material 4 presents a summary of the nonpowered explorations of effectiveness, which should be interpreted with caution.

#### Medication adherence

Data were unavailable for 2 of 3 medication adherence measures (NHS digital prescribing data and the MMAS-8) therefore the related progression criteria have been rated as amber. There were no notable trends for main effects of any component on medication adherence (assessed using the Voils DOSE) at 2 months. At 4 months, there was some evidence to suggest lower medication adherence among those who have received the information leaflet (adjusted mean difference = 0.088, 95% CI, 0.018, 0.158).

#### Quality of life

There were no trends for main effects of any components on the EQ-5D-5L summary score at 2 months. There was some evidence of a positive effect of the ACT component on the EQ-5D VAS summary score at 2 months (adjusted mean difference = 5.254, 95% CI, 1.285, 9.223). At 4 months, there was a trend for a positive effect of the leaflet component on the EQ-5D-5L summary score (adjusted mean difference = 0.038, 95% CI, −0.010, 0.085), but no trends for main effects of any component on the EQ-5D VAS score at 4 months.

There was a trend for a negative main effect of the website component on the McGill QoL score at 2 months (adjusted mean difference = −0.386, 95% CI, −0.832, 0.060). This was reversed at 4 months, where we observed trend for a positive main effect (adjusted mean difference = 0.311, 95% CI, −0.095, 0.716).

#### Symptoms and side effects

The website component was intended to target medication side effects and symptoms and therefore we specifically investigated the effect of this component on the EORTC QLQ-C30,-BR45, and IL133 measures. There was no notable trend for an effect of this component on the EORTC QLQ-C30 or BR45. There was some evidence of a positive effect of the website component on the IL133 vaginal discharge item at 4 months (adjusted mean difference = −4.689, 95% CI, −9.917, 0.539) and no trend at 2 months.

#### Medication beliefs

The information leaflet was intended to target medication beliefs. There was no trend for the main effect of this component on the BMQ-AET differential score at 2 months or 4 months.

#### Habits

The SMS component was intended to target medication-taking habits. There was a trend for a positive effect of the SMS component on SRBAI scores at 2 months (adjusted mean difference = 0.437, 95% CI, −0.019, 0.893), but not at 4 months.

#### Psychological distress

The ACT component was intended to target psychological distress. There was no trend for an effect of this component on DASS anxiety, depression, or stress scores at 2 months or 4 months.

#### Psychological flexibility

The ACT component was intended to target psychological flexibility. There was no trend for an effect of this component on the psychological flexibility or inflexibility subscales of the MPFI-SF at 2 months. At 4 months, there was a trend indicating higher psychological inflexibility among those receiving the ACT component (adjusted mean difference = 0.162, 95% CI, −0.036, 0.360), but no trend for psychological flexibility.

### Safety

No serious adverse events, related and unexpected serious adverse events, deaths, or pregnancies were reported during the trial.

## Discussion

In this pilot trial of a multicomponent intervention to support AET adherence in women with breast cancer, predefined criteria were met to support our decision to progress to a fully powered O-RCT. We were able to recruit participants at a sufficient rate, and an acceptable number of participants adhered to the minimal dose of the intervention anticipated to be sufficient to provide an effect. It was not feasible to collect data for 2 of our 3 medication adherence measures. After minor amendments, we are proceeding with a fully powered O-RCT with the self-reported Voils DOSE scale as the primary outcome measure as this has also been shown to be valid and reliable in other clinical contexts.^52,65^

This pilot trial provided useful data to inform the design and delivery of our future O-RCT. The trial experience data showed that no amendments to our data collection processes or study documents were necessary, as the majority of participants were satisfied with the information they were given, the time taken to participate in the trial and their overall experience. However, we acknowledge that the data provided by participants were retained within the trial to 4 months. Satisfaction among those who withdrew from different aspects of the trial was not investigated but is likely to have been different.

We observed a number of withdrawals from the SMS and ACT components. Informed by these data and those from our process evaluation involving acceptability questionnaires and semistructured interviews, we reviewed these components.^39^ For the SMS component, this included offering a preferred time of day for receiving messages and inviting a patient panel to review the message content further. For the ACT component, we have extended the period between therapy sessions from 1 to 2 weeks, provided more detailed information regarding what to expect from these sessions in the participant information sheet, and extended the duration of the first and final session from 15 to 25 min.

Three of our candidate intervention components were low-cost and if shown to be effective could be implemented in the healthcare system with relative ease. The ACT component was more expensive due to the involvement of highly trained clinical psychologists. While the value of supporting adherence to AET is significant,^10^ the financial cost of clinical psychologists is also high. We will explore the feasibility of including more affordable healthcare professionals, including Assistant Psychologists and Psychological Wellbeing Practitioners, in the future O-RCT, given the lower intensity and parsimony of the ACT intervention, combined with feedback from therapists suggesting such an approach warrants exploration. While cost-effectiveness analysis using data from factorial trials has its own challenges,^66^ we will consider how to include cost data into the objectives and analysis of the future O-RCT.

In underpowered exploratory analysis, there were relatively few signals of effectiveness for each of the 4 candidate intervention components on the planned primary outcome of medication adherence. This was also the case for each of the components on the predicted intervention targets. We reported some evidence of a negative effect of the information leaflet on medication adherence at 4-month follow-up. While we recommend little emphasis is placed on this finding due to lack of power, we will be able to investigate formally in the planned O-RCT. The 2^4-1^ fractional factorial design used here aliases main effects with interaction effects, making interpretation of interactions between components challenging. We will therefore include the information leaflet as a component in the planned O-RCT, as the leaflet could contribute positively to adherence via interactions with other intervention components, which we will be able to estimate in our planned O-RCT using a full factorial design.

This trial had limitations. While we were able to recruit at a sufficient rate, once eligible patients were identified, the initial process of identification took longer than expected and compared unfavorably with our earlier trial in a similar population.^67^ This led to a smaller sample size than we had planned, as we stopped recruiting at the end of our defined recruitment period. Compared with the eligible population of patients screened, our randomized sample was younger, more likely to be of White ethnicity, and more likely to have recently completed treatment. This should be considered when generalizing our data to the broader patient population.

In conclusion, based on predefined progression criteria regarding recruitment, adherence to intervention components and availability of outcome data, this pilot fractional factorial trial of a multicomponent intervention to support AET adherence in women with breast cancer indicates progression to a fully powered O-RCT is warranted.

## Supplementary Material

kaaf003_suppl_Supplementary_Materials_1

kaaf003_suppl_Supplementary_Materials_2

kaaf003_suppl_Supplementary_Materials_3

kaaf003_suppl_Supplementary_Materials_4

## Transparency statements

*Study registration*: Clinical trial registration number: ISRCTN10487576. *Analytic plan preregistration*: A detailed statistical analysis plan was prepared prior to analysis, but this was not preregistered. *Data availability*: Trial data was held securely at the University of Leeds CTRU, and operational processes were defined for the transfer, storage, restricted access, and disposal of personal information. Data will be shared for participants who have consented to use of their data for secondary research and will only be made available in such a way that recipients cannot identify individuals by any reasonable likely means. Data will be shared for projects that are in the public interest and compatible with the original purpose of the data processing. Data access requests should be made to CTRU-DataAccess@leeds.ac.uk. *Analytic code availability*: Analytic code may be shared as part of a data access request*. Materials availability*: Materials used to conduct the trial may be shared, however, these are currently being evaluated in a larger trial and are unlikely to be shared until the research is complete. Requests for materials should be made to the corresponding author.
