# Supplementary material for: Supporting endocrine therapy adherence in women with breast cancer: findings from the ROSETA pilot fractional factorial randomized trial
Source: Ann Behav Med. 2025 Jan 31;59(1):kaaf003. doi: 10.1093/abm/kaaf003 (PMC11783298; doi:10.1093/abm/kaaf003)

**Electronic Supplementary Material 1. Conceptual model of ROSETA intervention. Figure taken from Green et al.,**^26^


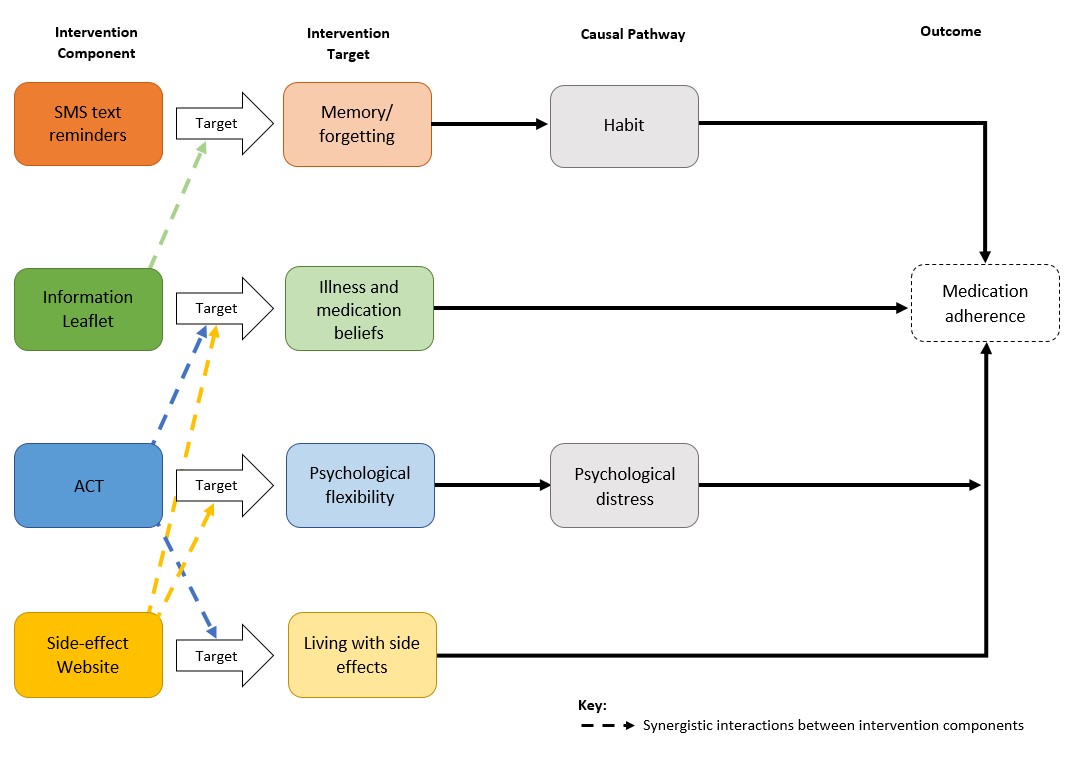

Supplement: kaaf003_suppl_Supplementary_Materials_1 [file kaaf003_suppl_supplementary_materials_1.docx]
