# Supplementary material for: Supporting endocrine therapy adherence in women with breast cancer: findings from the ROSETA pilot fractional factorial randomized trial
Source: Ann Behav Med. 2025 Jan 31;59(1):kaaf003. doi: 10.1093/abm/kaaf003 (PMC11783298; doi:10.1093/abm/kaaf003)
Supplement: kaaf003_suppl_Supplementary_Materials_2 [file kaaf003_suppl_supplementary_materials_2.docx]

**Supplement 2. Aliasing in the fractional factorial design as shown by colour coding**

| Condition | Usual care | Text reminders (T) | Information leaflet (L) | ACT (A) | Side-effect website (W) | T x L | T x A | T x W | L x A | L x W | W x A | T x L x A | L x A x W | T x L x W | T x A x W | T x L x A x W |
| --- | --- | --- | --- | --- | --- | --- | --- | --- | --- | --- | --- | --- | --- | --- | --- | --- |
| 1 | +1 | +1 | +1 | +1 | +1 | +1 | +1 | +1 | +1 | +1 | +1 | +1 | +1 | +1 | +1 | +1 |
| 2 | +1 | +1 | +1 | -1 | -1 | +1 | -1 | -1 | -1 | -1 | +1 | -1 | +1 | -1 | +1 | +1 |
| 3 | +1 | +1 | -1 | +1 | -1 | -1 | +1 | -1 | -1 | +1 | -1 | -1 | +1 | +1 | -1 | +1 |
| 4 | +1 | +1 | -1 | -1 | +1 | -1 | -1 | +1 | +1 | -1 | -1 | +1 | +1 | -1 | -1 | +1 |
| 5 | +1 | -1 | +1 | +1 | -1 | -1 | -1 | +1 | +1 | -1 | -1 | -1 | -1 | +1 | +1 | +1 |
| 6 | +1 | -1 | +1 | -1 | +1 | -1 | +1 | -1 | -1 | +1 | -1 | +1 | -1 | -1 | +1 | +1 |
| 7 | +1 | -1 | -1 | +1 | +1 | +1 | -1 | -1 | -1 | -1 | +1 | +1 | -1 | +1 | -1 | +1 |
| 8 | +1 | -1 | -1 | -1 | -1 | +1 | +1 | +1 | +1 | +1 | +1 | -1 | -1 | -1 | -1 | +1 |

Note: Identical colours in the columns above indicate where effects have been aliased. For example the 2-way interaction between text reminders and information leaflet is aliased with the 2-way interaction between the side-effect website and the ACT component (red columns).
