# Supplementary material for: Supporting endocrine therapy adherence in women with breast cancer: findings from the ROSETA pilot fractional factorial randomized trial
Source: Ann Behav Med. 2025 Jan 31;59(1):kaaf003. doi: 10.1093/abm/kaaf003 (PMC11783298; doi:10.1093/abm/kaaf003)
Supplement: kaaf003_suppl_Supplementary_Materials_3 [file kaaf003_suppl_supplementary_materials_3.docx]

| **Supplement 3. Characteristics of screened, eligible and randomized populations** | | | |
| --- | --- | --- | --- |
|  | **Screened**  **(N=175)** | **Eligible**  **(N=141)** | **Randomized (N=52)** |
| **Age** |  |  |  |
| Mean (SD) | 61.4 (12.8) | 60.9 (12.5) | 55.2 (10.8) |
| Missing | 23 | 3 | 0 |
| **Ethnicity** |  |  |  |
| White | 123 (79.9%) | 112 (80.0%) | 45 (86.5%) |
| Mixed | 4 (2.6%) | 3 (2.1%) | 2 (3.8%) |
| Asian | 5 (3.2%) | 5 (3.6%) | 2 (3.8%) |
| Black | 13 (8.4%) | 13 (9.3%) | 3 (5.8%) |
| Any other ethnic group | 1 (0.6%) | 1 (0.7%) | 0 (0.0%) |
| Not stated | 8 (5.2%) | 6 (4.3%) | 0 (0.0%) |
| Missing | 21 | 1 | 0 |
| **Stage of cancer at diagnosis** |  |  |  |
| Stage IA | 69 (47.6%) | 68 (49.3%) | 19 (38.0%) |
| Stage IB | 7 (4.8%) | 6 (4.3%) | 2 (4.0%) |
| Stage IIA | 35 (24.1%) | 33 (23.9%) | 15 (30.0%) |
| Stage IIB | 24 (16.6%) | 22 (15.9%) | 8 (16.0%) |
| Stage IIIA | 10 (6.9%) | 9 (6.5%) | 6 (12.0%) |
| Missing | 30 | 3 | 2 |
| **Tumor type** |  |  |  |
| Primary | 144 (95.4%) | 133 (95.7%) | 52 (100.0%) |
| Second primary | 4 (2.6%) | 4 (2.9%) | 0 (0.0%) |
| Recurrence | 3 (2.0%) | 2 (1.4%) | 0 (0.0%) |
| Missing | 24 | 2 | 0 (0.0%) |
| **Recruitment route** |  |  |  |
| Recently completed treatment | 108 (61.7%) | 82 (58.2%) | 36 (69.2%) |
| Self-referral | 10 (5.7%) | 10 (7.1%) | 9 (17.3%) |
| Retrospective screening | 57 (32.6%) | 49 (34.8%) | 7 (13.5%) |
