# Supplementary material for: Supporting endocrine therapy adherence in women with breast cancer: findings from the ROSETA pilot fractional factorial randomized trial
Source: Ann Behav Med. 2025 Jan 31;59(1):kaaf003. doi: 10.1093/abm/kaaf003 (PMC11783298; doi:10.1093/abm/kaaf003)
Supplement: kaaf003_suppl_Supplementary_Materials_4 [file kaaf003_suppl_supplementary_materials_4.docx]

**Supplement 4: Non-powered exploratory analysis**

| **Baseline summary scores of key participant outcome measures** | | | | | | | | |
| --- | --- | --- | --- | --- | --- | --- | --- | --- |
| **Outcome** | **SMS** | | **Leaflet** | | **ACT** | | **Website** | |
|  | **On** | **Off** | **On** | **Off** | **On** | **Off** | **On** | **Off** |
| **Voils DOSE-Extent Scale** | N=28 | N=24 | N=27 | N=25 | N=27 | N=25 | N=26 | N=26 |
| Mean (SD) | 1.14 (0.29) | 1.08 (0.30) | 1.12 (0.28) | 1.11 (0.32) | 1.10 (0.30) | 1.13 (0.29) | 1.13 (0.33) | 1.10 (0.26) |
| **EORTC QLQ-C30** |  |  |  |  |  |  |  |  |
| Global Health Status | N=28 | N=24 | N=27 | N=25 | N=27 | N=25 | N=26 | N=26 |
| *Mean (SD)* | 70.83 (17.05) | 60.42 (15.97) | 62.65 (19.66) | 69.67 (13.58) | 65.43 (18.01) | 66.67 (16.67) | 65.71 (16.39) | 66.35 (18.33) |
| **EORTC QLQ-BR45** |  |  |  |  |  |  |  |  |
| Endocrine therapy symptoms | N=28 | N=24 | N=27 | N=25 | N=27 | N=25 | N=26 | N=26 |
| *Mean (SD)* | 31.34 (23.06) | 42.14 (22.14) | 35.05 (22.93) | 37.70 (23.61) | 35.05 (22.50) | 37.70 (24.05) | 33.75 (21.08) | 38.90 (25.05) |
| **IL133** |  |  |  |  |  |  |  |  |
| Vaginal discharge | N=27 | N=24 | N=26 | N=25 | N=27 | N=24 | N=26 | N=25 |
| *Mean (SD)* | 9.88 (18.06) | 5.56 (12.69) | 6.41 (16.38) | 9.33 (15.28) | 7.41 (14.12) | 8.33 (17.72) | 6.41 (13.40) | 9.33 (18.05) |
| **EQ5D-5L** |  |  |  |  |  |  |  |  |
| Index | N=28 | N=24 | N=27 | N=25 | N=27 | N=25 | N=26 | N=26 |
| *Mean (SD)* | 0.78 (0.14) | 0.75 (0.11) | 0.75 (0.12) | 0.79 (0.14) | 0.77 (0.13) | 0.76 (0.13) | 0.74 (0.10) | 0.79 (0.15) |
| VAS | N=28 | N=24 | N=27 | N=25 | N=27 | N=25 | N=26 | N=26 |
| *Mean (SD)* | 71.07 (18.85) | 66.38 (18.53) | 65.74 (19.32) | 72.32 (17.68) | 68.63 (19.73) | 69.20 (17.85) | 69.62 (16.04) | 68.19 (21.27) |
| **MPFI** |  |  |  |  |  |  |  |  |
| Flexibility | N=28 | N=22 | N=25 | N=25 | N=26 | N=24 | N=25 | N=25 |
| *Mean (SD)* | 4.24 (0.94) | 4.06 (0.97) | 4.10 (0.90) | 4.21 (1.01) | 4.10 (0.93) | 4.23 (0.97) | 4.02 (0.78) | 4.30 (1.08) |
| Inflexibility | N=28 | N=23 | N=27 | N=24 | N=26 | N=25 | N=25 | N=26 |
| *Mean (SD)* | 2.40 (0.60) | 2.96 (0.79) | 2.62 (0.86) | 2.68 (0.59) | 2.63 (0.90) | 2.67 (0.56) | 2.60 (0.76) | 2.70 (0.74) |
| **BMQ-AET** |  |  |  |  |  |  |  |  |
| Differential | N=28 | N=24 | N=27 | N=25 | N=27 | N=25 | N=26 | N=26 |
| *Mean (SD)* | 5.93 (5.45) | 4.42 (6.37) | 4.33 (6.67) | 6.20 (4.84) | 6.30 (6.13) | 4.08 (5.50) | 6.73 (5.56) | 3.73 (5.92) |
| **SRBAI** | N=27 | N=23 | N=27 | N=23 | N=26 | N=24 | N=26 | N=24 |
| *Mean (SD)* | 4.82 (1.69) | 4.79 (1.70) | 5.00 (1.21) | 4.59 (2.10) | 4.88 (1.74) | 4.74 (1.64) | 4.69 (1.90) | 4.94 (1.42) |
| **DASS-21** |  |  |  |  |  |  |  |  |
| Stress (total) | N=28 | N=24 | N=27 | N=25 | N=27 | N=25 | N=26 | N=26 |
| *Mean (SD)* | 10.00 (6.55) | 15.42 (10.27) | 12.96 (8.40) | 12.00 (9.40) | 13.11 (9.79) | 11.84 (7.79) | 11.08 (8.91) | 13.92 (8.66) |
| Anxiety (total) | N=28 | N=24 | N=27 | N=25 | N=27 | N=25 | N=26 | N=26 |
| *Mean (SD)* | 4.43 (5.74) | 9.75 (10.57) | 5.26 (8.27) | 8.64 (8.90) | 6.74 (9.30) | 7.04 (8.11) | 5.85 (8.48) | 7.92 (8.88) |
| Depression (total) | N=28 | N=24 | N=27 | N=25 | N=27 | N=25 | N=26 | N=26 |
| *Mean (SD)* | 5.07 (5.87) | 9.75 (9.66) | 7.70 (8.80) | 6.72 (7.46) | 7.70 (9.52) | 6.72 (6.43) | 6.38 (7.18) | 8.08 (9.02) |
| **McGill QoL** |  |  |  |  |  |  |  |  |
| Overall | N=27 | N=24 | N=26 | N=25 | N=27 | N=24 | N=26 | N=25 |
| *Mean (SD)* | 7.42 (1.60) | 6.32 (1.66) | 6.86 (1.74) | 6.96 (1.70) | 6.89 (1.85) | 6.92 (1.56) | 6.85 (1.73) | 6.96 (1.71) |
| Note: The table shows raw summaries of participant -reported outcome measures, mean (SD). **Voils DOSE-Extent Scale**, range 1 (highest medication adherence) to 5 (lowest medication adherence). **EORTC QLQ-C30**= European Organization for Research and Treatment of Cancer Quality of Life Questionnaire, global health score status range 0 (worst health) to 100 (best health). **EORTC QLQ-BR45**= European Organization for Research and Treatment of Cancer Breast Cancer-Specific Quality of Life Questionnaire, endocrine therapy symptoms range 0 (highest level of problems) to 100 (lowest level of problems). **EQ-5D-5L** = European Quality of Life 5 Dimensions 5 Level Version, index score range -0.594 (worst health) to 1 (best health). **EQ-5D-5L VAS** = European Quality of Life 5 Dimensions 5 Level Version Visual Analogue Scale, range 0-100. **MPFI**= Multidimensional Psychological Flexibility Inventory, flexibility subscale range 0 (lowest level of flexibility) to 6 (highest level of flexibility), inflexibility subscale range 0 (lowest level of inflexibility) to 6 (highest level of inflexibility). **BMQ-AET**= Beliefs about Medicines Questionnaire- adjuvant endocrine therapy, differential score range -20 (most negative beliefs of medications) to 20 (most positive beliefs of medications). **SRBAI**= Self-Report Behavioural Automaticity Index, range 1 (weakest habits) to 7 (strongest habits). **DASS-21**= Depression Anxiety Stress Scales, range 0 (no stress/anxiety/depression) to 42 (extreme severe stress/anxiety/depression). **McGill QoL**= McGill Quality of Life, range 0 (worst situation) to 10 (best situation). | | | | | | | | |

| **Month 2 summary scores of key participant outcome measures** | | | | | | | | | |
| --- | --- | --- | --- | --- | --- | --- | --- | --- | --- |
| **Outcome** | **SMS** | | **Leaflet** | | **ACT** | | **Website** | |  |
|  | **On** | **Off** | **On** | **Off** | **On** | **Off** | **On** | **Off** |  |
| **Voils DOSE** | N=22 | N=21 | N=21 | N=22 | N=20 | N=23 | N=21 | N=22 |  |
| *Mean (SD)* | 1.23 (0.86) | 1.32 (0.90) | 1.27 (0.89) | 1.27 (0.87) | 1.32 (0.92) | 1.23 (0.84) | 1.24 (0.89) | 1.30 (0.87) |  |
| *Adjusted mean difference (95% CI)* | -0.061 (-0.324, 0.202) | | -0.037 (-0.298, 0.224) | | 0.040 (-0.221, 0.301) | | -0.012 (-0.276, 0.252) | |  |
| **EORTC QLQ-C30** |  |  |  |  |  |  |  |  |  |
| Global Health Status | N=22 | N=21 | N=21 | N=22 | N=20 | N=23 | N=21 | N=22 |  |
| *Mean (SD)* | 69.32 (14.64) | 66.27 (17.97) | 66.67 (16.03) | 68.94 (16.70) | 68.75 (16.42) | 67.03 (16.38) | 65.08 (14.82) | 70.45 (17.39) |  |
| *Adjusted mean difference (95% CI)* | -2.251 (-8.202, 3.701) | | 1.758 (-3.735, 7.250) | | 2.305 (-2.892, 7.503) | | -1.912 (-7.025, 3.201) | |  |
| **EORTC QLQ-BR45** |  |  |  |  |  |  |  |  |  |
| Endocrine therapy symptoms | N=22 | N=21 | N=21 | N=22 | N=20 | N=23 | N=21 | N=22 |  |
| *Mean (SD)* | 28.18 (22.22) | 38.75 (24.68) | 32.86 (21.79) | 33.80 (26.05) | 32.17 (24.60) | 34.36 (23.56) | 37.14 (23.93) | 29.71 (23.62) |  |
| *Adjusted mean difference (95% CI)* | 0.700 (-4.923, 6.323) | | 1.428 (-3.822, 6.677) | | 0.499 (-4.717, 5.714) | | 3.371 (-1.892, 8.635) | |  |
| **IL133** |  | |  | |  | |  | | |
| Vaginal discharge | N=22 | N=21 | N=21 | N=22 | N=20 | N=23 | N=21 | N=22 | |
| *Mean (SD)* | 10.61 (18.93) | 4.76 (11.95) | 7.94 (17.97) | 7.58 (14.30) | 11.67 (16.31) | 4.35 (15.26) | 4.76 (11.95) | 10.61 (18.93) | |
| *Adjusted mean difference (95% CI)* | 0.631 (-3.273, 4.535) | | 1.404 (-2.407, 5.215) | | 3.244 (-0.548, 7.037) | | -2.180 (-6.067, 1.708) | | |
| **EQ5D** |  |  |  |  |  |  |  |  |  |
| Index | N=22 | N=21 | N=21 | N=22 | N=20 | N=23 | N=21 | N=22 |  |
| *Mean (SD)* | 0.79 (0.12) | 0.75 (0.12) | 0.77 (0.13) | 0.77 (0.11) | 0.76 (0.12) | 0.78 (0.12) | 0.77 (0.13) | 0.78 (0.11) |  |
| *Adjusted mean difference (95% CI)* | 0.005 (-0.030, 0.039) | | 0.012 (-0.022, 0.046) | | -0.009 (-0.043, 0.024) | | 0.016 (-0.019, 0.051) | |  |
| VAS | N=22 | N=21 | N=21 | N=22 | N=20 | N=23 | N=21 | N=22 |  |
| Mean (SD) | 73.91 (15.30) | 71.48 (15.06) | 72.81 (15.34) | 72.64 (15.13) | 78.00 (12.95) | 68.13 (15.50) | 71.48 (11.59) | 73.91 (17.95) |  |
| Adjusted mean difference (95% CI) | -1.153 (-5.267, 2.961) | | 2.078 (-1.957, 6.114) | | 5.254 (1.285, 9.223) | | -1.660 (-5.670, 2.350) | |  |
| **MPFI** |  |  |  |  |  |  |  |  |  |
| Flexibility | N=21 | N=21 | N=20 | N=22 | N=20 | N=22 | N=21 | N=21 |  |
| *Mean (SD)* | 4.69 (0.70) | 4.15 (0.70) | 4.20 (0.77) | 4.62 (0.67) | 4.47 (0.79) | 4.38 (0.71) | 4.31 (0.75) | 4.54 (0.73) |  |
| *Adjusted mean difference (95% CI)* | 0.237 (0.002, 0.471) | | -0.195 (-0.425, 0.036) | | 0.004 (-0.227, 0.235) | | -0.067 (-0.301, 0.167) | |  |
| Inflexibility | N=22 | N=21 | N=21 | N=22 | N=20 | N=23 | N=21 | N=22 |  |
| *Mean (SD)* | 2.46 (0.46) | 3.06 (0.76) | 2.76 (0.59) | 2.75 (0.78) | 2.77 (0.73) | 2.74 (0.66) | 2.67 (0.71) | 2.83 (0.67) |  |
| *Adjusted mean difference (95% CI)* | -0.162 (-0.361, 0.036) | | 0.054 (-0.122, 0.231) | | -0.043 (-0.217, 0.130) | | -0.125 (-0.306, 0.056) | |  |
| **BMQ-AET** |  |  |  |  |  |  |  |  |  |
| Differential | N=22 | N=21 | N=21 | N=22 | N=20 | N=23 | N=21 | N=22 |  |
| *Mean (SD)* | 5.07 (5.89) | 4.29 (6.27) | 4.12 (7.19) | 5.23 (4.75) | 4.83 (5.72) | 4.57 (6.39) | 6.26 (5.60) | 3.18 (6.14) |  |
| *Adjusted mean difference (95% CI)* | -0.011 (-1.381, 1.359) | | 0.360 (-1.063, 1.783) | | -0.228 (-1.566, 1.109) | | 0.389 (-1.053, 1.831) | |  |
| **SRBAI** | N=22 | N=20 | N=21 | N=21 | N=20 | N=22 | N=21 | N=21 |  |
| *Mean (SD)* | 5.47 (1.26) | 4.60 (1.77) | 5.15 (1.41) | 4.95 (1.74) | 5.01 (1.89) | 5.09 (1.25) | 5.13 (1.52) | 4.98 (1.65) |  |
| *Adjusted mean difference (95% CI)* | 0.437 (-0.019, 0.893) | | 0.053 (-0.400, 0.506) | | -0.072 (-0.527, 0.383) | | 0.200 (-0.257, 0.657) | |  |
| **DASS-21** |  |  |  |  |  |  |  |  |  |
| Stress (total) | N=22 | N=21 | N=21 | N=22 | N=20 | N=23 | N=21 | N=22 |  |
| *Mean (SD)* | 11.18 (9.11) | 12.73 (8.44) | 14.19 (9.21) | 9.79 (7.83) | 12.60 (8.18) | 11.36 (9.30) | 11.43 (7.38) | 12.42 (9.98) |  |
| *Adjusted mean difference (95% CI)* | 1.395 (-0.881, 3.671) | | 1.672 (-0.378, 3.722) | | -0.083 (-2.166, 2.000) | | 0.664 (-1.461, 2.788) | |  |
| Anxiety (total) | N=22 | N=21 | N=21 | N=22 | N=20 | N=23 | N=21 | N=22 |  |
| *Mean (SD)* | 4.36 (5.00) | 9.40 (9.72) | 6.10 (7.28) | 7.52 (8.75) | 7.32 (8.19) | 6.39 (7.99) | 6.02 (7.02) | 7.59 (8.94) |  |
| *Adjusted mean difference (95% CI)* | 0.214 (-1.621, 2.049) | | 0.820 (-0.860, 2.501) | | -0.060 (-1.711, 1.592) | | -0.166 (-1.836, 1.504) | |  |
| Depression (total) | N=22 | N=21 | N=21 | N=22 | N=20 | N=23 | N=21 | N=22 |  |
| *Mean (SD)* | 5.55 (6.32) | 8.67 (10.01) | 8.19 (9.40) | 6.00 (7.33) | 7.00 (9.70) | 7.13 (7.26) | 6.57 (6.55) | 7.55 (9.95) |  |
| *Adjusted mean difference (95% CI)* | 0.785 (-1.701, 3.270) | | 0.336 (-1.924, 2.596) | | -0.960 (-3.255, 1.334) | | -0.060 (-2.323, 2.203) | |  |
| **McGill QoL** |  |  |  |  |  |  |  |  |  |
| Overall | N=22 | N=21 | N=21 | N=22 | N=20 | N=23 | N=21 | N=22 |  |
| *Mean (SD)* | 7.41 (1.57) | 6.50 (1.48) | 6.89 (1.38) | 7.04 (1.77) | 6.93 (1.72) | 7.00 (1.47) | 6.52 (1.59) | 7.39 (1.47) |  |
| *Adjusted mean difference (95% CI)* | 0.064 (-0.428, 0.555) | | 0.038 (-0.400, 0.475) | | 0.007 (-0.431, 0.445) | | -0.386 (-0.832, 0.060) | |  |
| Note: The table shows raw summaries of participant -reported outcome measures, mean (SD) and the half main effect of each component along with 95% confidence interval of the half main effect. | | | | | | | | |  |

| **Month 4 summary scores of key participant outcome measures** | | | | | | | | |
| --- | --- | --- | --- | --- | --- | --- | --- | --- |
| **Outcome** | **SMS** | | **Leaflet** | | **ACT** | | **Website** | |
|  | **On** | **Off** | **On** | **Off** | **On** | **Off** | **On** | **Off** |
| **Voils DOSE** | N=20 | N=21 | N=20 | N=21 | N=19 | N=22 | N=19 | N=22 |
| *Mean (SD)* | 1.15 (0.28) | 1.16 (0.27) | 1.25 (0.34) | 1.06 (0.13) | 1.16 (0.34) | 1.15 (0.20) | 1.12 (0.25) | 1.18 (0.29) |
| *Adjusted mean difference (95% CI)* | -0.027 (-0.098, 0.044) | | 0.088 (0.018, 0.158) | | 0.009 (-0.061, 0.079) | | -0.051 (-0.122, 0.021) | |
| **EORTC QLQ-C30** |  |  |  |  |  |  |  |  |
| Global Health Status | N=19 | N=20 | N=19 | N=20 | N=17 | N=22 | N=17 | N=22 |
| *Mean (SD)* | 68.42 (17.03) | 63.75 (16.06) | 64.91 (17.48) | 67.08 (15.87) | 63.24 (14.75) | 68.18 (17.75) | 68.14 (15.38) | 64.39 (17.48) |
| *Adjusted mean difference (95% CI)* | 2.980 (-4.151, 10.112) | | -0.209 (-6.571, 6.153) | | -1.999 (-8.229, 4.231) | | 3.452 (-2.512, 9.416) | |
| **EORTC QLQ-BR45** |  |  |  |  |  |  |  |  |
| Endocrine therapy symptoms | N=18 | N=20 | N=18 | N=20 | N=16 | N=22 | N=16 | N=22 |
| *Mean (SD)* | 23.89 (14.25) | 40.33 (26.90) | 27.96 (20.17) | 36.67 (25.27) | 34.17 (21.38) | 31.36 (24.72) | 36.25 (25.59) | 29.85 (21.34) |
| *Adjusted mean difference (95% CI)* | -1.916 (-8.000, 4.167) | | -1.117 (-6.750, 4.515) | | 2.019 (-3.405, 7.442) | | 0.990 (-4.482, 6.461) | |
| **IL133** |  |  |  |  |  |  |  |  |
| Vaginal discharge | N=18 | N=20 | N=18 | N=20 | N=16 | N=22 | N=16 | N=22 |
| *Mean (SD)* | 16.67 (23.57) | 6.67 (13.68) | 11.11 (19.80) | 11.67 (19.57) | 14.58 (20.97) | 9.09 (18.35) | 4.17 (11.39) | 16.67 (22.42) |
| *Adjusted mean difference (95% CI)* | 1.606 (-3.682, 6.894) | | 0.802(-4.499, 6.103) | | 2.132 (-2.989, 7.253) | | -4.689 (-9.917, 0.539) | |
| **EQ5D** |  |  |  |  |  |  |  |  |
| Index | N=20 | N=21 | N=20 | N=21 | N=19 | N=22 | N=19 | N=22 |
| *Mean (SD)* | 0.79 (0.12) | 0.73 (0.18) | 0.77 (0.12) | 0.75 (0.18) | 0.78 (0.13) | 0.74 (0.17) | 0.73 (0.10) | 0.78 (0.19) |
| *Adjusted mean difference (95% CI)* | 0.001 (-0.046, 0.049) | | 0.038 (-0.010, 0.085) | | 0.027 (-0.018, 0.073) | | 0.002 (-0.046, 0.051) | |
| VAS | N=20 | N=21 | N=20 | N=21 | N=19 | N=22 | N=19 | N=22 |
| Mean (SD) | 76.10 (15.32) | 67.81 (17.66) | 67.65 (18.00) | 75.86 (15.09) | 72.37 (18.70) | 71.41 (15.58) | 70.63 (15.52) | 72.91 (18.28) |
| Adjusted mean difference (95% CI) | 1.376 (-2.647, 5.400) | | -1.764 (-5.735, 2.207) | | 0.500 (-3.380, 4.379) | | -0.576 (-4.509, 3.356) | |
| **MPFI** |  |  |  |  |  |  |  |  |
| Flexibility | N=19 | N=21 | N=19 | N=21 | N=18 | N=22 | N=18 | N=22 |
| *Mean (SD)* | 4.23 (0.69) | 4.31 (0.97) | 4.11 (0.87) | 4.41 (0.81) | 4.25 (0.91) | 4.28 (0.80) | 4.31 (0.94) | 4.23 (0.77) |
| *Adjusted mean difference (95% CI)* | -0.042 (-0.308, 0.224) | | -0.117 (-0.375, 0.141) | | -0.025 (-0.283, 0.233) | | 0.155 (-0.111, 0.421) | |
| Inflexibility | N=19 | N=21 | N=19 | N=21 | N=18 | N=22 | N=18 | N=22 |
| *Mean (SD)* | 2.38 (0.41) | 3.05 (0.82) | 2.84 (0.84) | 2.63 (0.62) | 2.90 (0.72) | 2.59 (0.73) | 2.60 (0.70) | 2.84 (0.75) |
| *Adjusted mean difference (95% CI)* | -0.306 (-0.525, -0.087) | | 0.051 (-0.150, 0.252) | | 0.162 (-0.036, 0.360) | | -0.140 (-0.344 , 0.064) | |
| **BMQ-AET** |  |  |  |  |  |  |  |  |
| Differential | N=19 | N=20 | N=19 | N=20 | N=17 | N=22 | N=17 | N=22 |
| *Mean (SD)* | 4.71 (4.91) | 5.55 (5.08) | 4.68 (5.64) | 5.58 (4.30) | 5.82 (4.87) | 4.61 (5.06) | 7.56 (4.69) | 3.27 (4.38) |
| *Adjusted mean difference (95% CI)* | -0.785 (-2.049, 0.479) | | 0.241 (-1.032, 1.513) | | 0.621 (-0.587, 1.829) | | 1.240 (-0.047, 2.528) | |
| **SRBAI** | N=19 | N=19 | N=19 | N=19 | N=17 | N=21 | N=17 | N=21 |
| *Mean (SD)* | 5.42 (1.01) | 5.22 (1.88) | 5.32 (1.34) | 5.33 (1.68) | 5.54 (1.44) | 5.14 (1.55) | 5.51 (1.46) | 5.17 (1.54) |
| *Adjusted mean difference (95% CI)* | 0.176 (-0.279, 0.632) | | -0.132 (-0.580, 0.316) | | 0.352 (-0.090, 0.794) | | 0.461 (0.007, 0.916) | |
| **DASS-21** |  |  |  |  |  |  |  |  |
| Stress (total) | N=19 | N=19 | N=19 | N=19 | N=17 | N=21 | N=17 | N=21 |
| *Mean (SD)* | 8.61 (7.39) | 15.47 (10.41) | 12.30 (9.74) | 11.79 (9.64) | 12.71 (10.34) | 11.51 (9.10) | 10.94 (9.72) | 12.94 (9.56) |
| *Adjusted mean difference (95% CI)* | -1.336 (-4.626 , 1.955) | | 0.084 (-2.675, 2.843) | | -0.460 (-3.254, 2.333) | | -0.858 (-3.733 , 2.016) | |
| Anxiety (total) | N=19 | N=19 | N=19 | N=19 | N=17 | N=21 | N=17 | N=21 |
| *Mean (SD)* | 3.58 (3.86) | 11.37 (8.90) | 5.37 (6.53) | 9.58 (8.60) | 8.94 (8.92) | 6.29 (6.82) | 6.94 (8.55) | 7.90 (7.39) |
| *Adjusted mean difference (95% CI)* | -2.094 (-3.821 , -0.368) | | -0.726 (-2.297, 0.846) | | 0.654 (-0.820, 2.128) | | -0.715 (-2.222, 0.793) | |
| Depression (total) | N=19 | N=19 | N=19 | N=19 | N=17 | N=21 | N=17 | N=21 |
| *Mean (SD)* | 3.58 (4.35) | 7.93 (7.69) | 6.00 (6.80) | 5.51 (6.46) | 6.39 (7.79) | 5.24 (5.49) | 4.63 (5.48) | 6.67 (7.30) |
| *Adjusted mean difference (95% CI)* | -0.552 (-2.835, 1.731) | | -0.297 (-2.204 , 1.610) | | -0.444 (-2.408, 1.519) | | -1.155 (-3.096, 0.786) | |
| **McGill QoL** |  |  |  |  |  |  |  |  |
| Overall | N=18 | N=20 | N=18 | N=20 | N=17 | N=21 | N=17 | N=21 |
| *Mean (SD)* | 7.73 (1.26) | 6.57 (1.25) | 6.96 (1.26) | 7.26 (1.48) | 7.06 (1.21) | 7.17 (1.52) | 7.25 (1.37) | 7.02 (1.40) |
| *Adjusted mean difference (95% CI)* | 0.259 (-0.209, 0.728) | | -0.088 (-0.475, 0.300) | | 0.047 (-0.351, 0.446) | | 0.311 (-0.095, 0.716) | |
| Note: The table shows raw summaries of participant -reported outcome measures, mean (SD) and the half main effect of each component along with 95% confidence interval of the half main effect. | | | | | | | | |
